# Relationship between pyrimidines, purines, and fatty acids in milk of dairy cows fed distinct carbohydrate types: A metabolomic approach

**DOI:** 10.3168/jdsc.2024-0612

**Published:** 2024-10-22

**Authors:** Giulio Giagnoni, Martin Riis Weisbjerg, Michela Errico, Marco Lapris, Nina Aagaard Poulsen, Julia Prangchat Stub Thomsen, Antonio Gallo, Gabriele Rocchetti

**Affiliations:** 1 Department of Animal and Veterinary Sciences, AU Viborg–Research Centre Foulum, Aarhus University, DK 8830 Tjele, Denmark; 2 Department of Animal Science, Food and Nutrition, Università Cattolica del Sacro Cuore, Via Emilia Parmense 84, 29122 Piacenza, Italy; 3 Department of Food Science, Aarhus University, Agro Food Park 48, DK 8200 Aarhus N, Denmark

## Abstract

The aim of this research was to evaluate changes in the milk metabolome of dairy cows fed different carbohydrate types from silages and concentrates, with special focus on purines and pyrimidines, likely associated with the microbial activity in the rumen. Furthermore, the relationship between the milk fatty acids and pyrimidine and purine metabolism retrieved in milk was investigated for potential correlations between these 2 groups of potential milk biomarkers. Twenty-four lactating dairy cows were used in a crossover design, where each cow received 2 of 4 diets. Diets were formulated based on a 2 by 2 factorial arrangement, using either grass-clover or corn silage as a forage source, and barley or dried beet pulp in the concentrate fraction. Fatty acids and liquid chromatography-MS metabolome of the milk were analyzed. The milk metabolome and fatty acid concentration could differentiate the 4 diets used in the trial, whereas the milk metabolome alone could only differentiate the 2 silage types. Milk odd-chain fatty acids (C17:1 and C17:0) were correlated with pyrimidine and purine derivatives, with uric acid being significantly higher in milk samples associated with corn silage diets. Therefore, the results indicate potential differences in microbial protein synthesis between the diets, but a validation with actual ruminal microbial protein synthesis and investigation in digestive biofluids are necessary for further confirmation.

## Highlights

• Milk metabolome and fatty acids can differentiate dietary carbohydrate types.
• Proxies for microbial protein production can be useful for milk productivity.
• Such proxies could be from odd-chain fatty acids or pyrimidine/purine metabolism.
• Pyrimidine and purine metabolism products can identify dietary carbohydrate types.
• Pyrimidines and purines showed significant correlations with odd-chain fatty acids.

## Graphical Abstract

**Figure:**
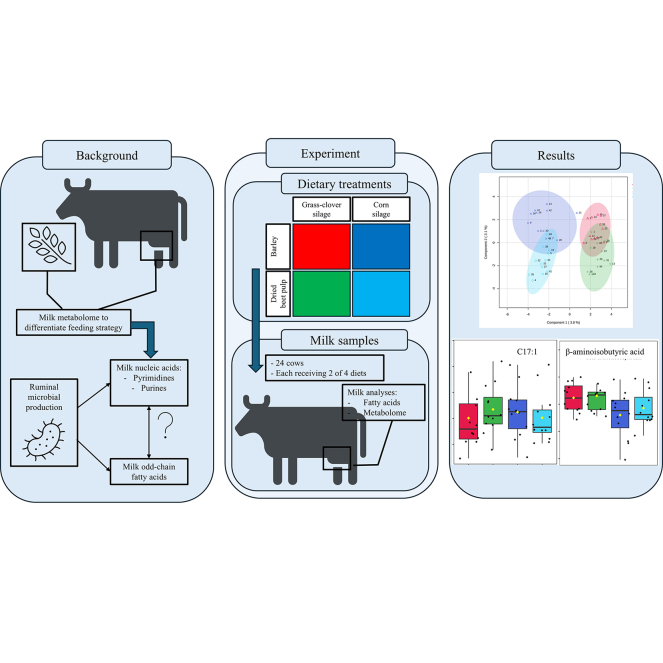
**Summary:** Diet can affect ruminal microbial protein production. Ruminal microbial protein production could be estimated by noninvasive methods, such as analysis of milk fatty acids or milk metabolome, but how do milk odd-chain fatty acids, a proxy for microbial protein production, correlate with milk compounds from pyrimidine and purine metabolism? In a crossover trial using different carbohydrate types, milk from dairy cows was analyzed for metabolomic profile and fatty acid concentration. The milk metabolome and fatty acid concentration could differentiate the 4 diets used in the trial, whereas the milk metabolome alone could only differentiate the 2 silage types. Some milk odd-chain fatty acids were correlated with pyrimidine and purine derivatives. Our results indicate that the milk metabolome could provide an indication of the protein efficiency of the diet; however, validation of the metabolomic findings with actual ruminal microbial protein synthesis data is necessary.

The diet of dairy cows can affect milk components such as the milk fatty acid (**FA**) profile (Slots et al., 2009; Coppa et al., 2015), as well as other compounds found in the milk metabolome (Zhu et al., 2021). Optimization of feeding in dairy cows is important to maximize feeding efficiency and minimize climate and environmental impact of dairy production. Protein supply is crucial to maximize feeding efficiency in dairy cows, and the ruminal microbial protein synthesis is important for the supply of protein for milk production. The production of rumen microbial protein is dependent on the nutrients supplied to the cow, and on the interaction between them (Firkins et al., 1998). However, measuring the production of rumen microbial protein requires invasive sampling methods such as intestinal cannulas or oro-ruminal sampling, which should be limited to a small number of animals. In contrast, milk samples can be collected and analyzed from a large number of individual cows on dairy farms. Milk FA have been used to predict rumen microbial production through the odd-chain FA (**OCFA**) found in the milk (Vlaeminck et al., 2005). Untargeted metabolomic analysis of milk samples is able to provide additional insight when compared with targeted FA analyses, and is able to differentiate between different diets (Rocchetti et al., 2022). However, the potential of milk metabolome to understand rumen microbial production has yet to be explored. The aim of this research was to evaluate changes in the milk metabolome of dairy cows fed different carbohydrate types from silages and concentrates, with special focus on the degradation products of nucleic acids, purine and pyrimidine, likely associated with the microbial activity in the rumen. Furthermore, the relationship between the milk FA and the products of the pyrimidine and purine metabolism retrieved in milk was investigated for potential correlations between these 2 groups of potential milk biomarkers. The full details regarding the experimental protocol are available in Giagnoni et al. (2024). Twenty-four lactating Danish Holstein cows were used in a crossover design with 2 periods, where cows were fed 2 of 4 diets. The allocation of the diets was made to balance for the carry-over effect so all possible combination of 2 diets were represented equally; more details on the design can be found in Giagnoni et al. (2024). The diets (55% of forage, % of DM) were factorially arranged 2 by 2, with either grass-clover or corn silage (**GRS** or **CS**, respectively) as the sole forage source, and either barley or dried beet pulp (**BAR** or **DBP**, respectively) included as 21% of DM. The rest of the diet included protein and other ingredients to fulfill the milk production requirement. In CS diets 8 g/kg (DM basis) of corn silage was substituted with a urea mix (80% urea) to balance the CP content between diets. Three milk samples were collected in the morning milking from individual cows in each period on d 19 and used for further analyses.

The extraction of milk metabolites was carried out as previously reported in Rocchetti et al. (2022). The collected milk samples (a total of 48 samples) were skimmed by centrifugation at 4,500 × *g* for 10 min at 4°C and the skim milk frozen at −80°C for further processing. Samples were thawed and thoroughly vortex-mixed. A 2-mL aliquot was extracted with 14 mL of acetonitrile (liquid chromatography-MS grade, Sigma-Aldrich) added with 3% formic acid (vol/vol), mixed by vortexing for 3 min, and processed with ultrasounds for 5 min. Milk samples were then centrifuged at 12,000 × *g* for 15 min at 4°C to enhance protein precipitation. The supernatants were filtered through 0.22-μm cellulose syringe filters in ultra-HPLC (**UHPLC**) vials until further untargeted metabolomic profiling. The untargeted UHPLC with high-resolution mass spectrometry (**HRMS**) analysis was done using a Q-Exactive Focus Hybrid Quadrupole-Orbitrap Mass Spectrometer (Thermo Scientific) coupled to a Vanquish UHPLC pump and equipped with heated electrospray ionization-II probe (Thermo Scientific). All the details related to chromatography and MS conditions are described in Rocchetti et al. (2022). The collected raw mass spectral data were processed using the MS-DIAL software (version 4.90), and the annotation was done via spectral matching against the Bovine Metabolome Database (https://bovinedb.ca/), FooDB (https://foodb.ca/), and Milk Composition Database (https://mcdb.ca/). Accurate mass tolerance for identification was 0.05 and 0.1 Da for MS1 and MS/MS, respectively. The identification step was based on mass accuracy, isotopic pattern, and spectral matching, considering a 5 ppm tolerance for mass accuracy. The most important compounds associated with pyrimidine and purine metabolic pathways (Rocchetti et al., 2022) were then quantified using dedicated UHPLC-HRMS conditions against authentic standard compounds of β-aminoisobutyric acid, allantoin, and uric acid (all from Sigma-Aldrich). Calibration curves were built using linear fitting in the range of 0.025 to 1 mg/L; a coefficient of determination higher than 0.98 was used as an acceptability threshold for calibration purposes. The milk samples used for FA analysis were centrifuged (2,643 × *g* at 4°C for 30 min), and the fat part removed from the skim milk was stored at −20°C. The fat was analyzed using GC like previously reported by Larsen et al. (2013), with the difference that heptane was used as solvent instead of pentane. After GC separation, peak areas for individual FA were calculated. External standards (FIM-FAME-7 mixture, Matreya Inc., PA, and CLA standard Sigma, Poole, UK) were used to identify and quantify the FA. The FA profile was expressed as grams per 100 g of total milk FA. The third milk sample was analyzed for urea at Eurofins laboratory (Vejen, Denmark) with a MilkoScan 7RM FT+ (Foss, Hillerød, Denmark). The omics dataset was elaborated for multivariate statistical modeling using the software MetaboAnalyst 6.0, as previously reported (Rocchetti et al., 2022). Data were median-centered, Pareto-scaled, and log_2_-transformed before building unsupervised and supervised models, both unsupervised and supervised multivariate statistics were carried out. The unsupervised approach was based on hierarchical cluster analysis, while the sparseness partial least squares discriminant analysis (**sPLS-DA**) and orthogonal projections to latent structures discriminant analysis (**OPLS-DA**) were used as supervised tools. Additionally, the OPLS-DA model validation parameters (goodness-of-fitting R^2^ together with goodness-of-prediction) were inspected, considering a goodness-of-prediction >0.5 as the acceptability threshold. Analysis of variance multiblock partial least squares was used to check significant interactions between silage and concentrate types. The importance of each milk metabolite for discriminating “GRS vs. CS” was calculated using the variable importance in projection (**VIP**) approach, considering as the minimum significant threshold with a score >1. Volcano plots, coupling fold-change (cutoff value >1.2) and ANOVA (*P* < 0.05), were used to inspect the statistical significance of each VIP marker compound. Finally, Pearson correlation coefficients (*P* < 0.05) between some potential milk biomarkers (i.e., purines and pyrimidines) and OCFA were calculated using the software SPSS (version 26.0).

The untargeted UHPLC-HRMS based approach allowed the identification of 539 milk metabolites according to a level 2 of confidence in annotation (Blaženović et al., 2018). In addition, the analysis of pooled quality control samples provided the structural identification of 134 compounds against the spectral information of the comprehensive Bovine Metabolome Database. A following enrichment analysis on MetaboAnalyst 6.0 allowed us to group the most enriched metabolites of the omics dataset. These latter belonged to the main classes of amino acids and peptides (46 key compounds), followed by pyrimidines (14 key compounds), purines (8 key compounds), and lipid derivatives (>50 key metabolites, mainly belonging to fatty esters and glycerophospholipids). Interestingly, among the most abundant compounds revealed by UHPLC-HRMS through pooled quality controls (supplemental material, see Notes), we found 4 metabolites, namely, β-aminoisobutyric acid (i.e., a thymidine degradation product previously reported to be associated with rumen activity in dairy cows; Rocchetti et al., 2022), β-guanidinopropionic acid (i.e., an organonitrogen compound belonging to the class of guanidines, being an analog of creatine and involved in energy metabolism; Oudman et al., 2013), glucocapparin (i.e., belonging to glucosinolates and appearing in milk as the result of transferred phenomena from forages and concentrates; EFSA, 2008), and pristanal (i.e., an aldehyde deriving from the metabolism of phytanic acid from chlorophyll; Gutbrod et al., 2021). To check the impact of the nutritional strategies on the milk metabolome and milk FA we first carried out an unsupervised clustering analysis (not shown) highlighting high variability between the different sample groups. Therefore, a supervised sPLS-DA score plot (not shown) was used to better evaluate the hierarchical weight of the silage type (GRS vs. CS) when comparing the milk from cows fed BAR and DBP diets. The sPLS-DA score plot (not shown) outlined a higher discriminant weight for the silage type, driving the modification of milk metabolome, although a certain within-group variation could be measured as well by the supervised algorithm. Since no significant interactions (*P* > 0.05) between silage and concentrate types were found using ANOVA multiblock PLS approach (not shown), we studied the only main effect, dealing with the comparison of the 2 silage types.

Two additional supervised OPLS-DA prediction models were then built by splitting the variability given by the 2 dietary factors, and considering the following pairwise comparisons, namely “GRS vs. CS” and “BAR vs. BRP” (Figure 1A and Figure 1B). Overall, the first OPLS prediction model (Figure 1A) was characterized by almost acceptable goodness-of-fitting and goodness-of-prediction parameters (being 0.986 and 0.420, respectively); on the other hand, the second OPLS prediction model (Figure 1B) showed only a good fitting ability (R^2^ = 0.951) but no accuracy in terms of prediction, thus confirming the higher robustness of the milk metabolites annotated for discriminating mainly the 2 different silage types, as provided by ANOVA multiblock PLS. Nevertheless, given its different supervised nature, OPLS-DA allowed us to extrapolate the discriminant compounds for each comparison under investigation by using a VIP selection method. The discrimination between “GRS vs. CS” diets revealed a total of 170 metabolites owning a VIP score >1, whereas the second model discriminating “BAR vs. DPB” (although showing no significant prediction ability) provided 171 VIP compounds. Thereafter, the utilization of a Venn diagram (not shown) allowed us to separate both common and exclusive metabolites of each pairwise comparison, revealing 81 exclusive compounds dealing with the comparison “GRS vs. CS.” To strengthen the discriminant power of the VIP metabolites identified, we considered only those compounds also showing a significant variation as resulting from the volcano plot analysis (i.e., combination of ANOVA and fold-change analyses), owning a VIP score >2.5. Some of these discriminant metabolites are finally reported in Table 1 for both the considered comparisons.

**Figure 1:**
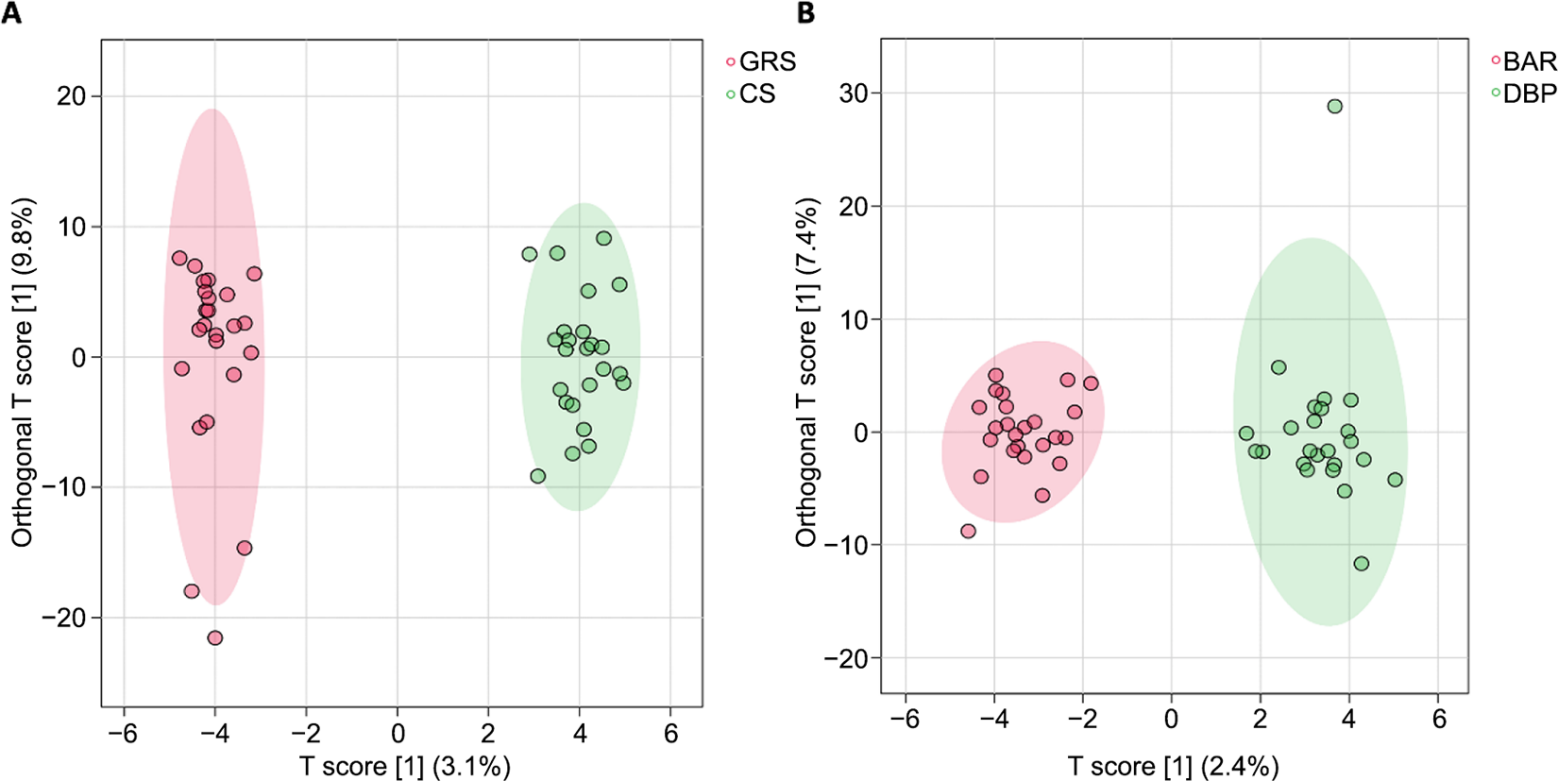
Score plots obtained from orthogonal projections to latent structures discriminant analyses built considering the milk metabolites and milk fatty acids for the pairwise comparisons grass-clover versus corn silage (A; GRS vs. CS) and barley versus dried beet pulp (B; BAR versus DBP).

**Table 1:** Some discriminant milk metabolites of the pairwise comparisons “GRS vs. CS” and “BAR vs. BRP”

Discriminant metabolite	VIP1^1^	VIP2^1^	VIP (total)^1^	Log_2_ FC^2^	*P*-value
GRS vs. CS^3^					
Resveratrol	5.30	0.12	5.42	5.59	<0.01
9-Oxo-octadecanoic acid	3.96	0.83	4.79	−1.42	<0.01
Isopalmitic acid	4.36	0.26	4.61	−1.69	<0.01
7-Methylxanthine	1.52	2.35	3.87	0.74	<0.05
Orotidine	1.73	1.82	3.55	0.64	<0.05
Uric acid	2.67	0.49	3.16	−0.32	<0.01
α-Tocotrienol	1.72	1.13	2.85	1.26	<0.05
β-Aminoisobutyric acid	2.12	0.74	2.85	0.29	<0.05
BAR vs. BRP^3^					
Cer(d18:0/24:0)	2.31	1.58	3.89	−0.41	<0.05
PC[20:1(11Z)/22:1(13Z)]^4^	2.18	1.71	3.89	−0.32	<0.05
PC(20:0/24:0)^4^	2.57	1.22	3.79	−0.77	<0.01
Xanthosine 5-triphosphate	2.31	1.48	3.79	−0.28	<0.05
Spermic acid	2.37	0.68	3.05	0.57	<0.05
Vulgaxanthin II	2.34	0.37	2.71	−0.28	<0.05

1 Total variable importance in projection (VIP) score (which resulted from the prediction ability between and within groups, being VIP1 and VIP2, respectively).

2 Log_2_ fold-change.

3 GRS = grass-clover silage; CS = corn silage; BAR = barley; DBP = dried beet pulp.

4 PC = phosphatidylcholine; PE = phosphatidylethanolamine.

Overall, 38 compounds were identified as potential milk biomarkers of the model “GRS vs. CS”; in particular, isopalmitic acid and 9-oxo-octadecanoic acid were the most promising predictive compounds of CS diets, while among the most significant compounds of GRS diets we found typical antioxidant and bioactive compounds, such as resveratrol (belonging to stilbenes) and α-tocotrienol. Additionally, the untargeted metabolomics approach allowed the identification of 16 compounds described to be involved in the degradation pathway of pyrimidines because of rumen microbial activity (Stentoft et al., 2015; Rocchetti et al., 2022). In particular, we found metabolites related to both cytosine and thymine biochemical pathways, namely 2′-deoxycytidine, 2′-deoxyuridine, β-alanine, β-aminoisobutyric acid, β-ureidoisobutyric acid, β-ureidopropionic acid, cytidine 5′-monophosphate, 2′-deoxycytidine 5′-monophosphate, cytidine, cytosine, dihydrothymine, dihydrouracil, 2′-deoxythymidine 5′-monophosphate (**dTMP**), 2′-deoxyuridine 5′-monophosphate, uridine 5′-monophosphate, and thymidine (supplemental material, see Notes). Additionally, as far as the purine degradation pathway is concerned, we found again several metabolites belonging to this biochemical pathway, such as allantoin, allantoic acid, uric acid, methylated forms of guanine and hypoxanthine, and both nucleosides and nucleotide (including derivatives of adenosine and guanosine). Uric acid (from purine degradation pathway) and β-aminoisobutyric acid (from pyrimidine degradation pathway) were included among the most discriminant and predictive compounds of GRS versus CS comparison (Table 1).

Interestingly, as reported in Table 2, the Pearson correlation coefficients allowed us to observe a hierarchical higher importance (in terms of significant correlations) of pyrimidine derivatives with the milk FA previously determined in Giagnoni et al. (2024). Four metabolites (i.e., 2′-deoxycytidine, 2′-deoxyuridine, β-aminoisobutyric acid, and dTMP, characterizing the degradation pathway of pyrimidines) showed significant and negative correlation coefficients with OCFA, whereas the only metabolite from purine degradation pathway establishing significant correlations was allantoic acid (i.e., representing the hydrolytic product of allantoin, previously described in literature as an potential biomarker for rumen microbial synthesis; Gonda and Lindberg, 1997). Therefore, the low but significant correlation coefficients between some pyrimidine degradation products and OCFA allowed us to postulate that these compounds are likely involved in the complex metabolism of microbial nitrogen based on total splanchnic fluxes from the rumen to mammary gland in dairy cows. Accordingly, the same pyrimidine degradation products were used in a previous work (Rocchetti et al., 2022) to trace different feeding strategies, being the marker compounds in milk of high-moisture corn intake by dairy cows.

**Table 2:** Pearson correlation coefficients between the most discriminant milk metabolites (from purine and pyrimidine degradation pathways) and fatty acid profile

Fatty acid	2'-Deoxycytidine	2'-Deoxyuridine	β-Aminoisobutyric acid	dTMP^1^	Allantoic acid
C11:0	−0.401**	−0.407**	NS	NS	NS
C13:0	−0.442**	−0.446**	NS	NS	NS
C15:0	−0.353*	−0.366*	NS	−0.382**	NS
C17:1	NS	NS	−0.447**	NS	0.289*
C17:0	NS	NS	NS	NS	0.385**
Total OCFA^2^	−0.337*	−0.375**	NS	−0.373**	NS
C4:0	0.334*	NS	0.376**	NS	NS
C6:0	NS	NS	0.561**	0.363*	NS
C8:0	NS	NS	0.571**	0.456**	NS
C10:0	NS	NS	0.298*	0.366*	NS

1 dTMP = 2′-deoxythymidine 5′-monophosphate.

2 OCFA = odd-chain fatty acids.

* *P < 0.01,

** *P* < 0.05; NS = not significant (*P* > 0.05).

Fatty acids C15:0 and C17:0 are produced from 2-carbon elongation of propionate (Xin et al., 2021). Therefore, it is not surprising that several studies have reported close relationships between OCFA and ruminal VFA composition both in vitro and in vivo (Vlaeminck et al., 2006; French et al., 2012). As far as the quantification of key metabolites of purines and pyrimidines is concerned, β-aminoisobutyric acid showed a not significant (*P* > 0.05) variation between GRS (on average: 120.6 mg/L) and CS (on average: 101.3 mg/L), as resulting from one-way ANOVA. Also, we found no significant differences for allantoin (on average: 0.24 mg/L for all milk samples tested), whereas an opposite trend was outlined for uric acid. Uric acid was significantly up accumulated in milk samples belonging to CS diets (on average: 2.43 mg/L) when compared with GRS diets (on average: 1.95 mg/L). Overall, allantoin and uric acid are associated with a high xanthine oxidase activity in the blood and tissues, converting xanthine and hypoxanthine into uric acid before excretion. It is known that CS is a high-energy forage that promotes efficient microbial growth in the rumen (Miguel et al., 2021). This can enhance the synthesis of microbial CP, which, when degraded, increases the production of purine derivatives like uric acid, thus reflecting improved nitrogen utilization and protein metabolism in the rumen. These purine degradation products have been previously proposed as indirect markers of rumen microbial synthesis (Schager et al., 2003). Also, some previous works suggest that milk uric acid may be modulated by ruminal nitrogen metabolism and DMI (Larsen and Moyes, 2010; Billa et al., 2020). In our trial, CS diets were supplemented with urea, but CS-BAR diet also had higher DMI and CP intake. Either of these factors, the urea supplementation or the CP intake, might have driven the difference in milk uric acids between CS and GRS diets. No correlation was found between milk urea and the main products of purine catabolism, namely uric acid, allantoic acid, and allantoin. Both serve as important indicators of nitrogen balance and metabolic health, but they originate from different biochemical pathways. It cannot be excluded that degradation products from the pyrimidine and purine metabolism are affected by the microbiome of the silage, but to investigate such relationship these compounds should be followed from the feedstuff to the digestive metabolism, and finally in the excretion products. In this regard, our work shows how some compounds are able to partially discriminate feeding strategies through untargeted metabolomics; however, a better understanding of the absorbed quantity and intermediate metabolism of purines and pyrimidines could be given through targeted metabolomics analyses, not only in milk but also in other biofluids, such as plasma, urine, rumen, and feces. Finally, the potential correlation between milk uric acid and microbial CP in the rumen should be mentioned (Lima et al., 2023) as a valuable indicator for managing and optimizing protein nutrition in dairy cows. Further research and practical applications of this relationship can lead to improved feeding strategies and more efficient dairy production systems.
